# The Corset Once More

**Published:** 1891-06

**Authors:** 


### THE CORSET ONCE MORE.

Elizabeth Stuart Phelps Ward, whose pen adds interest to any subject, says this of the corset:

Had I the sole responsibility of dressing a more or less stylish young lady in some approach to a sensible manner, I would begin by removing her corsets.

Of course she "never laces." Of course she can "turn around in them." We know all about that. We have heard it a great many times before. It is a very old story. If a woman were dying of the close clasp of steel and whalebone, she would cry, "Loose enough!" with her last gasp. Undoubtedly she believes it—more's the pity! We will not pause to argue the point with her. Off with the corsets!

Take them down stairs. No, don't give them to Biddy. Never fasten about another woman, in the sacred name of charity, the chains from which you have yourself escaped. What is intrinsically unbecoming or unrighteous, is as unbecoming or unrighteous for your cook as for yourself. So burn up the corsets! No, nor do you save the whalebones. You will never need whalebones again. Make a bonfire of the cruel steel that has lorded it over the contents of the abdomen and thorax so many thoughtless years, and heave a sigh of relief; for your "emancipation," I assure you, has from this moment begun.

A certain sense of freedom follows this change. The lungs partially dilate. The heart feebly feels for bounding room. The nerve centres are disturbed with an uncertain parody of ease. But a greater sense of discomfort grows upon you. The back, perhaps for the first time in your life, begins to ache. The spine grows sore to the touch. An exhausting faintness takes possession of you. The delicate sensibilities of the solar plexus (let us be learned when we can!) quiver in an irritated and irritating manner. A worrying nervousness takes possession of the whole frame. By night you are ready to resurrect the ashes of your departed corsets—for which I was quite prepared when I begged for their *auto-da-fe*. You are miserable and discouraged. This is precisely the point at which most women who "mean well" are led to abandon their half-hearted or half-instructed efforts at dress reform.

So far from indicating that you should return to your stays, these uncomfortable sensations indicate that you have not been a day too soon in their removal. These are not the sensations of a healthy and untrammeled organism. The uncorseted savage knows nothing of them. Are women born in whalebone jackets? Did Heaven create Eve with a natural inability to hold her fair, fresh body up without the assistance of Mrs. Ford's latest patent? Is there reason, in the eternal nature of things, why your brother can stand straight, and feel at ease, in clothing as loose as your wrapper, and you "drop all together," unless you can lean upon a long steel rod?

Your discomfort is the discomfort of the poor old prisoner in the "Tale of Two Cities," who must needs bear with him, into his late and affluent liberty, the shoemaker's tools—the degrading sign of that sadder mental captivity of which the body's bondage was the type. Your sensations are the sensations of a released captive—not to be humored, be assured, for liberty's sweet sake. Every one of them is an appeal to you, in the name of common nature and of common sense, to persist in the unshackling which has produced them.

But as time wears on, you grow more and more uncomfortable. It occurs to you one day that your bindings are too tight. Of course they are. So is your dress waist. You loosen the bindings an inch, another, two ; the demands of the partially released and ever struggling organs behind them grow, and will not be satisfied. Some idea of the unnatural restriction to which the ordinary modes of dress subject a woman's system, may be formed from the fact that a woman who now wears a twenty-two inch corset, will require, at the end of one year and a half of corsetless existence, a thirty-inch dress binding for her comfortable wear.

Loosen you bindings, then, till freed nature is content. You will have a more graceful figure in the end, than ever you have had yet.

